# Gadolinium oxide nanocrystal nonvolatile memory with HfO_2_/Al_2_O_3 _nanostructure tunneling layers

**DOI:** 10.1186/1556-276X-7-177

**Published:** 2012-03-08

**Authors:** Jer-Chyi Wang, Chih-Ting Lin, Chia-Hsin Chen

**Affiliations:** 1 Department of Electronic Engineering, Chang Gung University, No. 259, Wen-Hua 1st Road, Kwei-Shan, Tao-Yuan, 333, Taiwan, Republic of China

**Keywords:** NVMs, Gd_2_O_3_, nanocrystal, nanostructure, HfO_2_/Al_2_O_3_, tunneling layer

## Abstract

In this study, Gd_2_O_3 _nanocrystal (Gd_2_O_3_-NC) memories with nanostructure tunneling layers are fabricated to examine their performance. A higher programming speed for Gd_2_O_3_-NC memories with nanostructure tunneling layers is obtained when compared with that of memories using a single tunneling layer. A longer data retention (< 15% charge loss after 10^4 ^s) is also observed. This is due to the increased physical thickness of the nanostructure tunneling layer. The activation energy of charge loss at different temperatures is estimated. The higher activation energy value (0.13 to 0.17 eV) observed at the initial charge loss stage is attributed to the thermionic emission mechanism, while the lower one (0.07 to 0.08 eV) observed at the later charge loss stage is attributed to the direct tunneling mechanism. Gd_2_O_3_-NC memories with nanostructure tunneling layers can be operated without degradation over several operation cycles. Such NC structures could potentially be used in future nonvolatile memory applications.

## Introduction

Nanocrystal (NC) memory has been widely studied as a possible solution to the scaling-down problem that traditional floating gate (FG) nonvolatile memories (NVMs) have faced. It is believed that NC memory is superior to FG memories because of either the lower leakage current from the NCs to the Si substrate or the lower lateral electron migration between NCs [1-3]. In this regard, the tunneling oxide thickness can be reduced due to the enhancement of immunity against local oxide defects, thereby allowing higher charge injection efficiency through the tunneling oxide to the charge trapping layer. The performance of NC memory depends on the densities, sizes, and shapes of the NCs. Several NC materials such as silicon (Si), germanium (Ge), gold (Au), and platinum (Pt) have been used in memory devices [4-7]. Several approaches have been investigated in order to fabricate NCs. Among these, a common method is the use of a thermal annealing process to induce crystalline phase separation (such as in HfO_2_-NC) or condensation effects (Au-NC formation) [8-12]. However, the method of HfO_2_-NC formation requires a dual sputtering process, i.e., the Si and Hf targets are loaded simultaneously in an ambient argon and oxygen mixture to form a HfSiO layer; this is followed by rapid thermal annealing (RTA) treatment [8,9]. The Au-NC embedded in a SiO_2 _matrix is formed by annealing a Au thin film whose thickness is controlled to within 3 nm. The size and density of Au-NC are sensitive to the thickness of the Au thin film and the annealing temperature. This will lead to variations in the process control of Au-NC formation [10,11]. In recent years, the use of gadolinium oxide (Gd_2_O_3_) has attracted considerable attention for application as high-*k *gate dielectrics in complementary metal-oxide-semiconductor (CMOS) technologies [13]. Furthermore, the Gd_2_O_3 _was also demonstrated to be the potential candidate of III-V CMOS application because the trivalent oxide can be allowed to have a charge matching with the GaAs interface [14]. In addition, a few studies have demonstrated a method of synthesizing Gd_2_O_3_-NC via a few chemical reaction steps [15]. The simplest way to form Gd_2_O_3_-NC is the use of RTA treatment on an amorphous Gd_2_O_3 _(a-Gd_2_O_3_) thin film prepared by sputtering [16,17]. This method has been applied in memory fabrication; large memory windows and good data retention can be achieved by using optimized RTA temperatures [16]. Some parts of a-Gd_2_O_3 _will transform into a nanostructure crystalline phase after RTA treatment, while other parts remain in the amorphous phase. This procedure can natively form Gd_2_O_3_-NC embedded in an a-Gd_2_O_3 _thin film. Here, the smaller bandgap of Gd_2_O_3_-NC, which is surrounded by the larger bandgap of a-Gd_2_O_3_, could be responsible for the charge storage mechanism due to the bandgap offset [16,18].

Another solution to the scaling-down problem of NVMs is to substitute band-engineering silicon-oxide-nitride-oxide-silicon (BE-SONOS) for FG memories [19-21]. A Si_3_N_4 _film is treated as the charge-trapping layer in the BE-SONOS structure due to the presence of a large amount of discrete trap distributions, while the SiO_2_/SiN_x_/SiO_2 _layer is treated as the tunneling layer by exploiting the unique band structure and the increased physical thickness [19]. It has been demonstrated that BE-SONOS memories exhibit a good performance in terms of programming and erasing (P/E) speed and data retention. Further, high-*k *materials such as HfO_2 _have been applied to the tunneling oxide layer of NC memory because of their lower capacitance-equivalent thickness and lower band offset with Si substrates [22]. In this study, a nanostructure using a-Gd_2_O_3_/HfO_2_/Al_2_O_3 _as the tunneling layer is applied to Gd_2_O_3_-NC memories, in which a-Gd_2_O_3 _is a part of the Gd_2_O_3 _thin film. The HfO_2 _and Al_2_O_3 _layers were prepared by atomic layer deposition and radio frequency (RF) sputtering system, respectively. Data retention can be improved due to the increased physical thickness of the tunneling layer, and the P/E speed can be improved due to band alignment in the programming and erasing states.

## Experimental process

**Figure 1:**
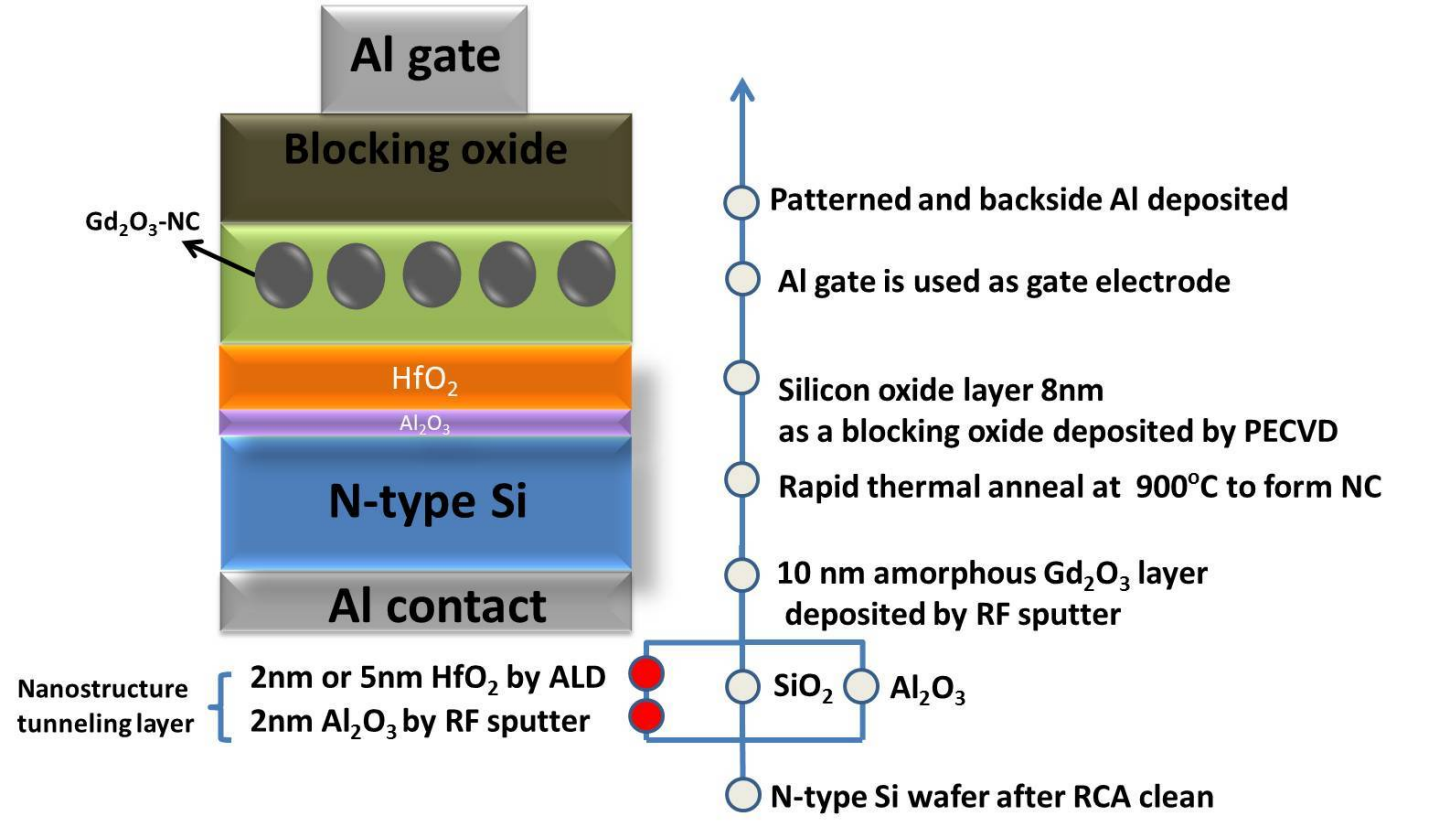
**The schematic structure of Gd_2_O_3_-NC memories with nanostructure tunneling layer**. There are four samples in this experiment. Two samples for the nanostructure HfO_2_/Al_2_O_3 _layer with 2 and 5 nm HfO_2_, respectively. Two samples for the SiO_2 _and Al_2_O_3 _single tunneling layers, respectively.

Figure 1 shows the schematic of Gd_2_O_3_-NC memories and the process flow involved in the fabrication. These devices were fabricated on 4-in., n-type (100) silicon wafers. After performing a wafer cleaning process, an Al_2_O_3_/HfO_2 _nanostructure tunneling layer was deposited. The Al_2_O_3 _layer was deposited via RF sputtering in an atmosphere consisting of an argon and oxygen mixture using a pure Al target (99.999% pure, ADMAT Inc., Norristown, PA, USA) as a source, while the HfO_2 _layer was deposited via an atomic layer deposition technique by using the tetrakis(ethylmethylamido)hafnium (Nammat Technology Co. Ltd., Kaohsiung, Taiwan, Republic of China) as a precursor. Two thicknesses of the HfO_2 _layer, 2 and 5 nm, are deposited for comparison and denoted as samples DL_1 (2 nm) and DL_2 (5 nm), respectively. Some of the samples used a grown SiO_2 _film or a deposited Al_2_O_3 _film as the single tunneling oxide layer. The splits of samples of different tunneling layer structures for comparative study are labeled in Table 1. Subsequently, a 10-nm-thick Gd_2_O_3 _layer was deposited on all samples by RF sputtering using a pure Gd target (99.9% pure, ADMAT Inc., Norristown, PA, USA) in an ambient argon and oxygen mixture in which the pressure of the gases was 20 mTorr. The flow ratio of argon to oxygen was 7:1. After forming the Gd_2_O_3 _layer, all of the samples underwent RTA at 900°C for 30 s in ambient nitrogen to form the Gd_2_O_3_-NC [16]. Some portions of the Gd_2_O_3 _were crystallized to form nanocrystals, while other portions formed a surrounding layer of a-Gd_2_O_3_. Subsequently, an 8-nm-thick SiO_2 _layer (as the blocking oxide) was deposited in an ambient SiH_4 _and N_2_O mixture at 300°C by a plasma-enhanced chemical vapor deposition technique. A 300-nm-thick Al film was deposited using a thermal coater with a pure Al ingot (99.9999% pure, ADMAT Inc., Norristown, PA, USA), and a gate was defined lithographically and etched to be the circle gate pattern with a diameter of 180 μm. In addition, an Al/HfO_2_/Al_2_O_3_/SiO_2_/Si capacitor was fabricated to monitor the characteristics of the device. For electrical analysis, the capacitance-voltage (C-V) hysteresis profile and the P/E characteristics were measured using Agilent 4284A precision LCR meter and 8110A pulse generator, respectively (Agilent Technologies, Inc., Santa Clara, CA, USA).

**Table 1:** Splits of samples of different tunneling layer structures for comparative study

Sample names	DL_1 (2 nm)	DL_2 (5 nm)	SiO_2_	Al_2_O_3_
Tunneling layer structure				

## Results and discussion

**Figure 2:**
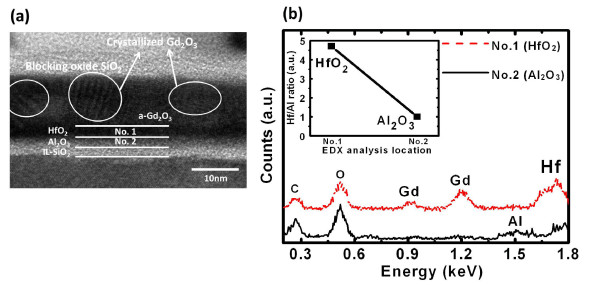
**HRTEM image and EDX analysis**. (**a**) HRTEM image of Gd_2_O_3_-NC memories with nanostructure tunneling layer. The marks No. 1 and No. 2 indicate the X-ray spot locations of the EDX analysis. (**b**) EDX analysis of locations No. 1 and No. 2 in the HRTEM image. Inset is the Hf/Al ratio of the two locations. The Gd_2_O_3_-NC embedded in a-Gd_2_O_3 _is observed in the HRTEM. The interfacial layer SiO_2 _is also observed between Al_2_O_3 _and Si.

**Figure 3:**
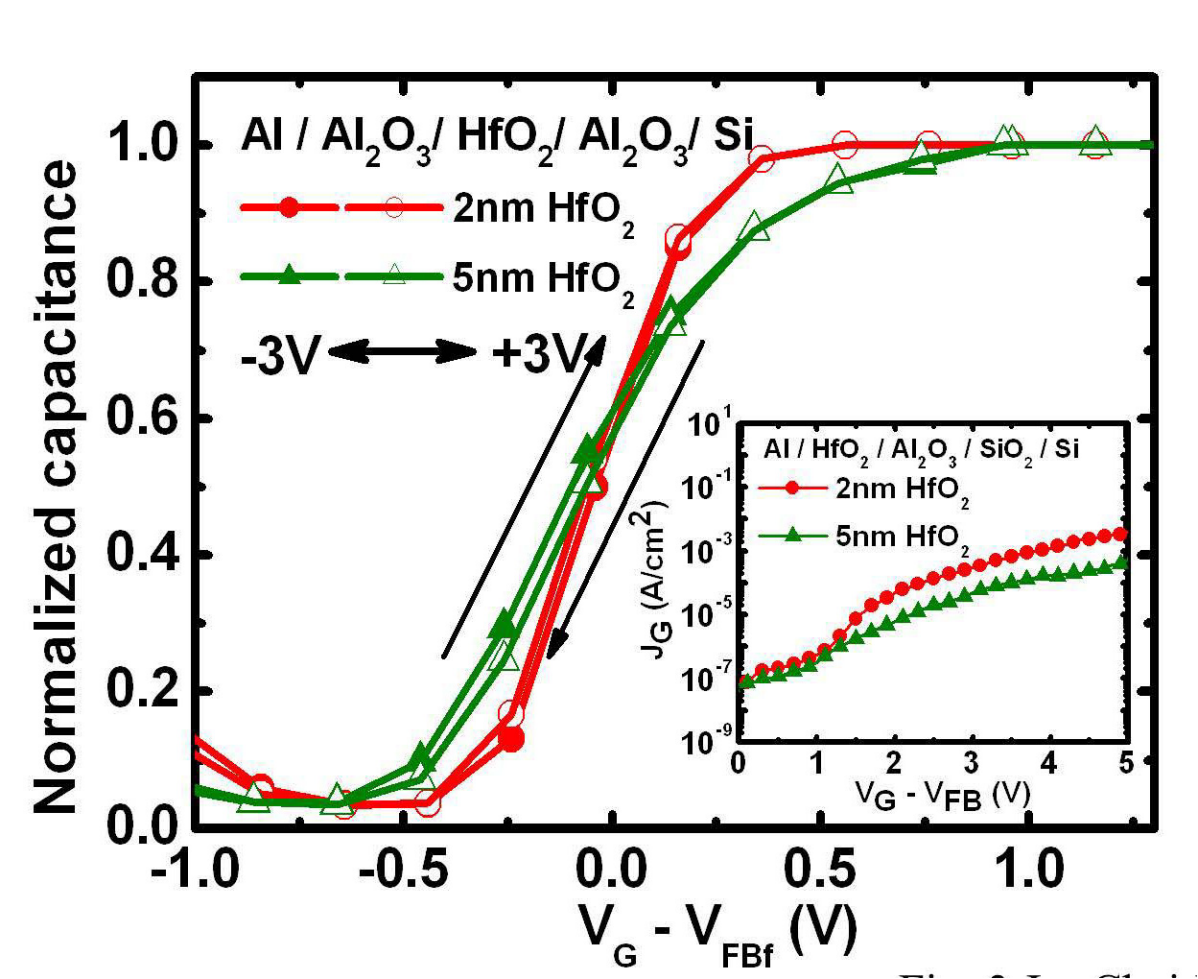
**The C-V hysteresis of capacitor structure Al/HfO_2_/Al_2_O_3_/SiO_2_/Si for two different HfO_2 _thicknesses**. Inset shows the J-V characteristic of the same capacitor structure. The gate voltage of the C-V hysteresis was swept from -3 to +3 V and then swept back. All the gate voltages were normalized with the *V*_FB _of the forward (-3 to +3V) C-V curve (*V*_FBf_).

**Figure 4:**
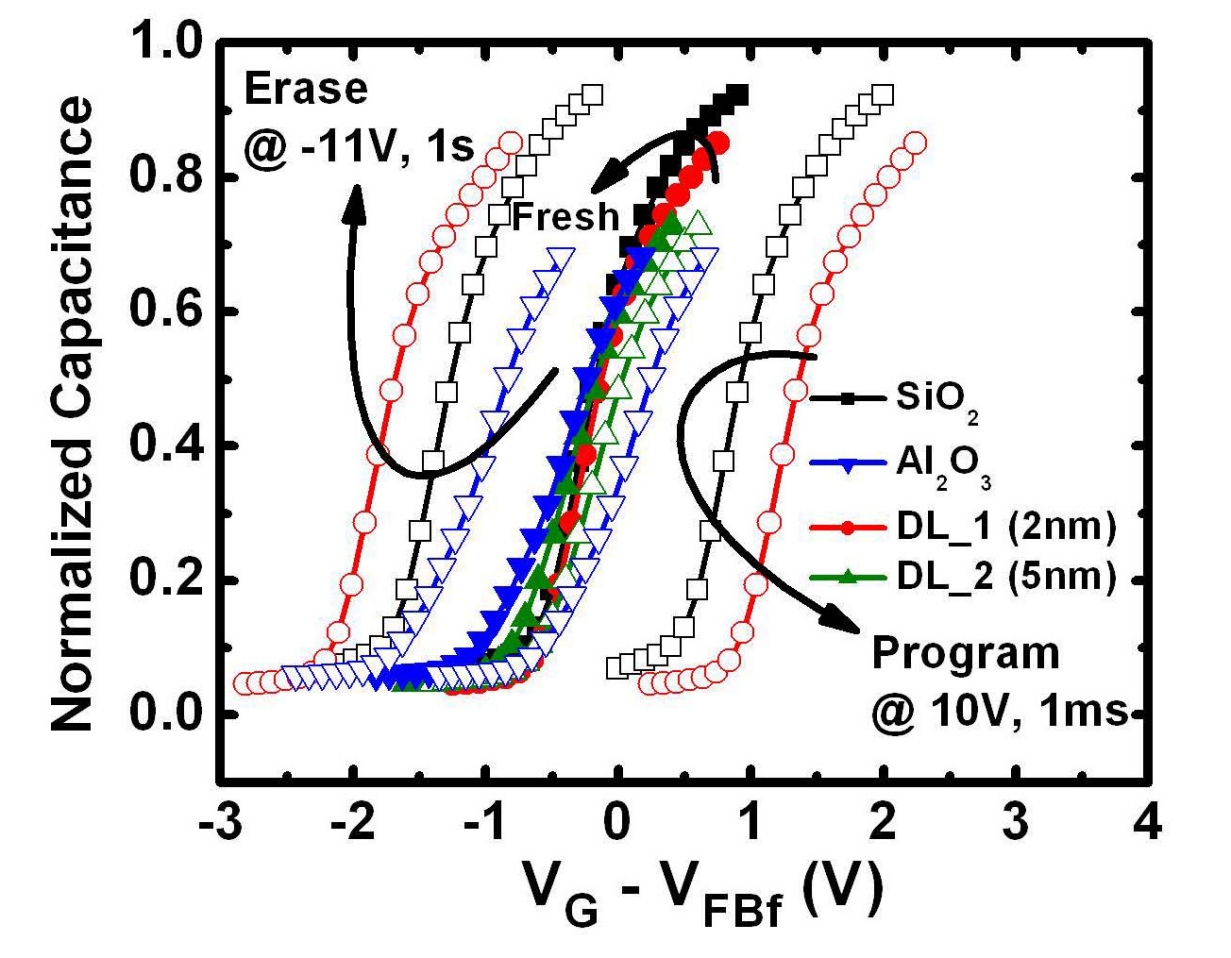
**The C-V curves**. The C-V curves of the fresh, programming (at 10 V, 1 ms) and erasing (at 11 V, 1 s) states for all samples. All the gate voltages were normalized with the *V*_FB _of the fresh-state C-V curve (*V*_FBf_).

**Figure 5:**
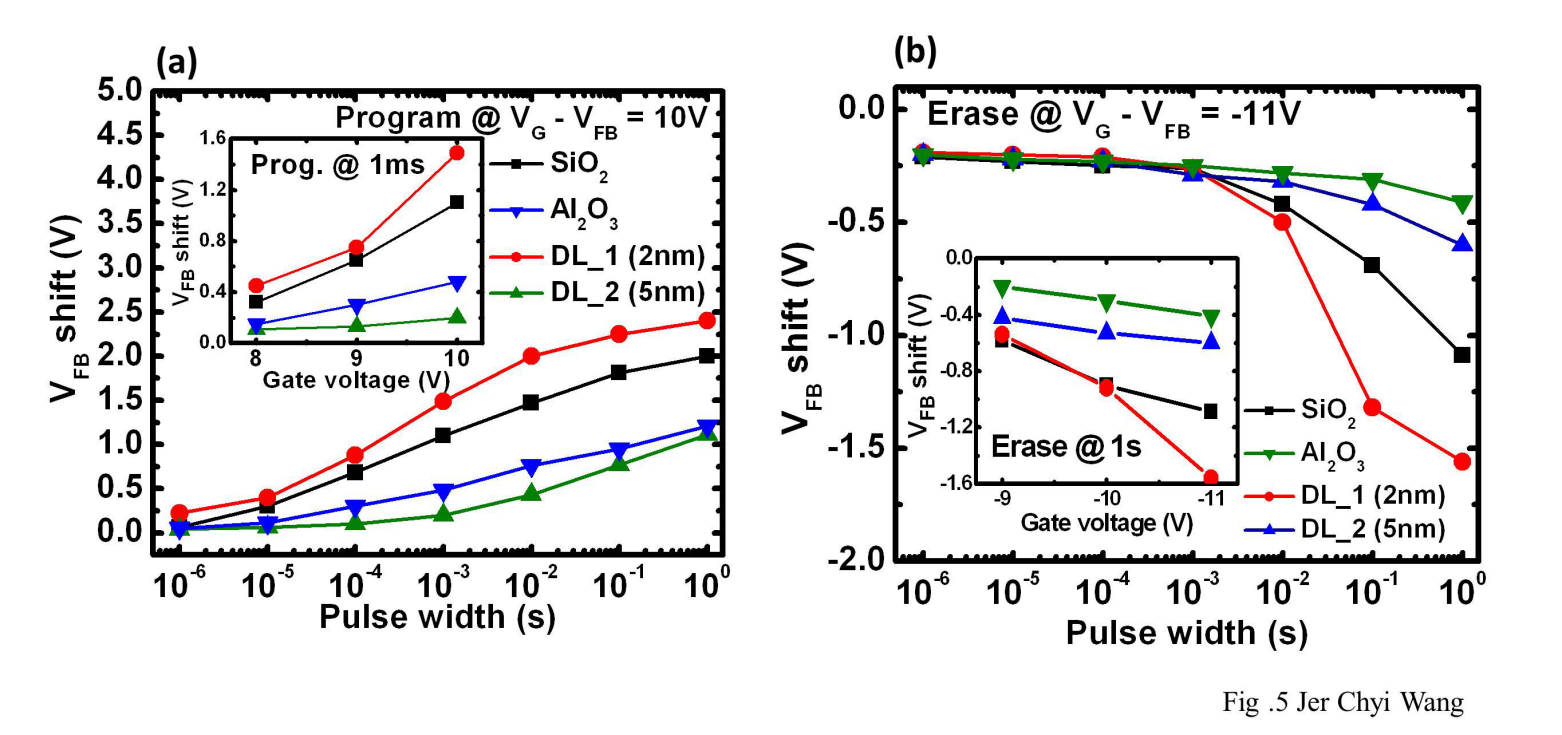
**Programming (a) and erasing (b) characteristic of Gd_2_O_3_-NC memories with nanostructure tunneling layer**. Insets show the extracted *V*_FB _shift of various P/E voltages at 1 ms/1 s.

Figure 2a shows the high-resolution transmission electron microscopy (HRTEM) image of the Gd_2_O_3_-NC memory structure in which the HfO_2 _layer is 2-nm thick (DL_1 (2 nm) sample). The crystallized Gd_2_O_3_-NC embedded in a-Gd_2_O_3 _that is observed is identical with that obtained in our previous study [16]. However, an interfacial layer of SiO_2_, with a thickness of about 2 nm, is also observed between the Al_2_O_3 _layer and the Si substrate. Figure 2b shows the energy-dispersive X-ray (EDX) analysis of the HfO_2 _and Al_2_O_3 _layers for which the spot locations of X-ray are pointed out in Figure 2a as 'No. 1' and 'No. 2', respectively. The Hf/Al ratio can be estimated using the highest counts of Hf and Al; this is shown in the inset of Figure 2b. A higher ratio is observed at location No. 1; this indicates that the HfO_2 _layer was formed on the Al_2_O_3 _layer. Figure 3 shows the C-V hysteresis of the capacitor nanostructure comprising Al/HfO_2_/Al_2_O_3_/SiO_2_/Si. Negligible hysteresis is obtained for both 2-nm HfO_2 _and 5-nm HfO_2_, thereby indicating that it is almost trap-free in the nanostructure tunneling layer. The inset in Figure 3 shows the gate current density versus gate voltage of this structure. It is observed that the gate current density of the structure with 2-nm HfO_2 _is higher than that of the structure with the thicker HfO_2 _layer. The application of the former nanostructure can improve the P/E efficiency of the Gd_2_O_3_-NC memory. The C-V curves of the fresh, programming, and erasing states of the Gd_2_O_3_-NC memories are shown in Figure 4. All the gate voltages were normalized with the flat-band voltage of the forward (negative to positive gate voltage) C-V curves (*V*_FBf_), and the capacitance values were normalized with oxide capacitance (*C*_ox_). The *V*_FB _shift in the P/E operations can be extracted from this figure. The P/E speeds are shown in Figure 5a, b, respectively. The gate voltage (*V*_G_) was set to (10 + *V*_FB_) V for the programming state and (-11 + *V*_FB_) V for the erasing state. The insets in Figure 5a, b show the extracted *V*_FB _shift for various programming and erasing gate voltages, respectively. The higher *V*_FB _shift for the DL_1 (2 nm) sample when compared with that for a single tunneling layer (SiO_2 _or Al_2_O_3_) can be observed. This could be due to the band alignment of the nanostructure tunneling layer when the gate voltage is being applied. This will be discussed later in the following text. On the other hand, the small *V*_FB _shift for the DL_2 (5 nm) sample could be due to the thicker HfO_2 _layer in the nanostructure tunneling layer. Detailed discussions regarding this *V*_FB _shift are to be described later in this paper.

**Figure 6:**
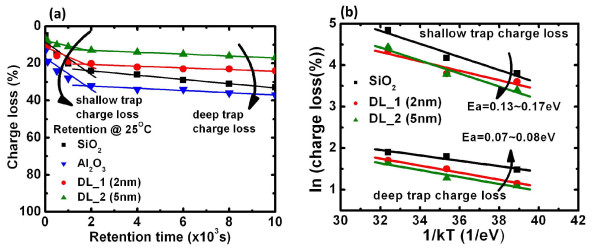
**Retention characteristic and extracted activation energy**. (**a**) The retention characteristic at 25°C of Gd_2_O_3_-NC memories with nanostructure tunneling layer. (**b**) The extracted activation energy of two charge loss mechanisms. The retention characteristic can be divided into two parts which have different charge loss rates.

The retention characteristics are shown in Figure 6a. The charge loss can be calculated by

(1) $$Q_{\text{loss}}\left(\%\right)\;=\;\frac{\left(V_{\text{FBp}}-V_{\text{FBt}}\right)}{\left(V_{\text{FBp}}-V_{\text{FBi}}\right)}\times \text{1}00\%,$$

where *V*_FBi _is the *V*_FB _of the initial memory status, *V*_FBp _is the *V*_FB _after programming, and *V*_FBt _is the flat-band voltage after the retention time. Thus, the charge loss rate can be given as $\frac{Q_{\text{loss}}}{\text{Δ}t}$, i.e., the tangent slope of charge loss versus retention time. In general, this retention curve can be approximately divided into two sections that have different charge loss rates. A higher initial charge loss rate is observed between 0 and 2,000 s, while a lower charge loss rate is observed between 2,000 and 10,000 s. In a previous study, it was reported that the higher charge loss rate in the initial stage is associated with the higher activation energy (*E*_a_) due to the charge loss from the shallow traps via the thermionic emission mechanism, while the charge loss rate in the later stage is associated with the lower activation energy due to the charge loss from the deep traps via the direct tunneling mechanism [23]. The lowest initial charge loss rate for DL_2 (5 nm) samples can be obtained since the physical thickness of the nanostructure tunneling layer is greater than that of the other samples. On the other hand, the initial charge loss rate of the sample with the Al_2_O_3 _tunneling layer is higher not only due to the reduced physical thickness, but also due to the lower conduction band offset between Al_2_O_3 _and Si [24]. In addition, the activation energy was extracted in order to understand the temperature dependence of the charge loss mechanism; this is shown in Figure 6b. The activation energy is determined using the relationship between charge loss and temperature, which is given as follows:

(2) $$Q_{\text{loss}}\propto \text{exp}\left(\frac{-E_{\text{a}}}{k_{\text{B}}T}\right).$$

Here, *Q*_loss _denotes the charge loss from the shallow-trap and deep-trap electron loss for the Gd_2_O_3_-NC memories, *E*_a _represents the activation energy for charge loss, *k*_B _denotes the Boltzmann constant, and *T *denotes the absolute temperature. The *E*_a _of the shallow-trap charge loss (0.13 to 0.17 eV) is higher than that of the deep-trap charge loss (0.07 to 0.08 eV). This indicates that the charge loss mechanism in the shallow trap is thermionic emission (which has higher dependence on temperature) while the charge loss mechanism in the deep trap is direct tunneling (lower temperature dependence). The charge loss mechanism in this case is identical with that reported before [23,25].

**Figure 7:**
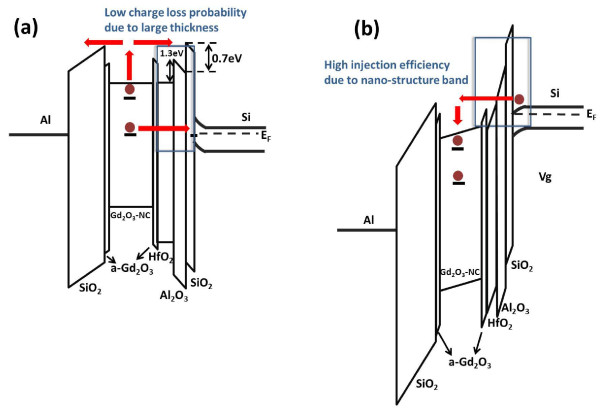
**The band diagrams of Gd_2_O_3_-NC memories with nanostructure at (a) retention and (b) programming states**. The bandgaps of HfO_2 _and Al_2_O_3 _are assumed to be 6.1 and 8.7 eV, respectively [22,23]. The charge loss paths of shallow traps and deep traps are pointed out by arrow signs in (a); the charge injection paths when applying gate voltage is drawn by arrow signs in (b).

**Figure 8:**
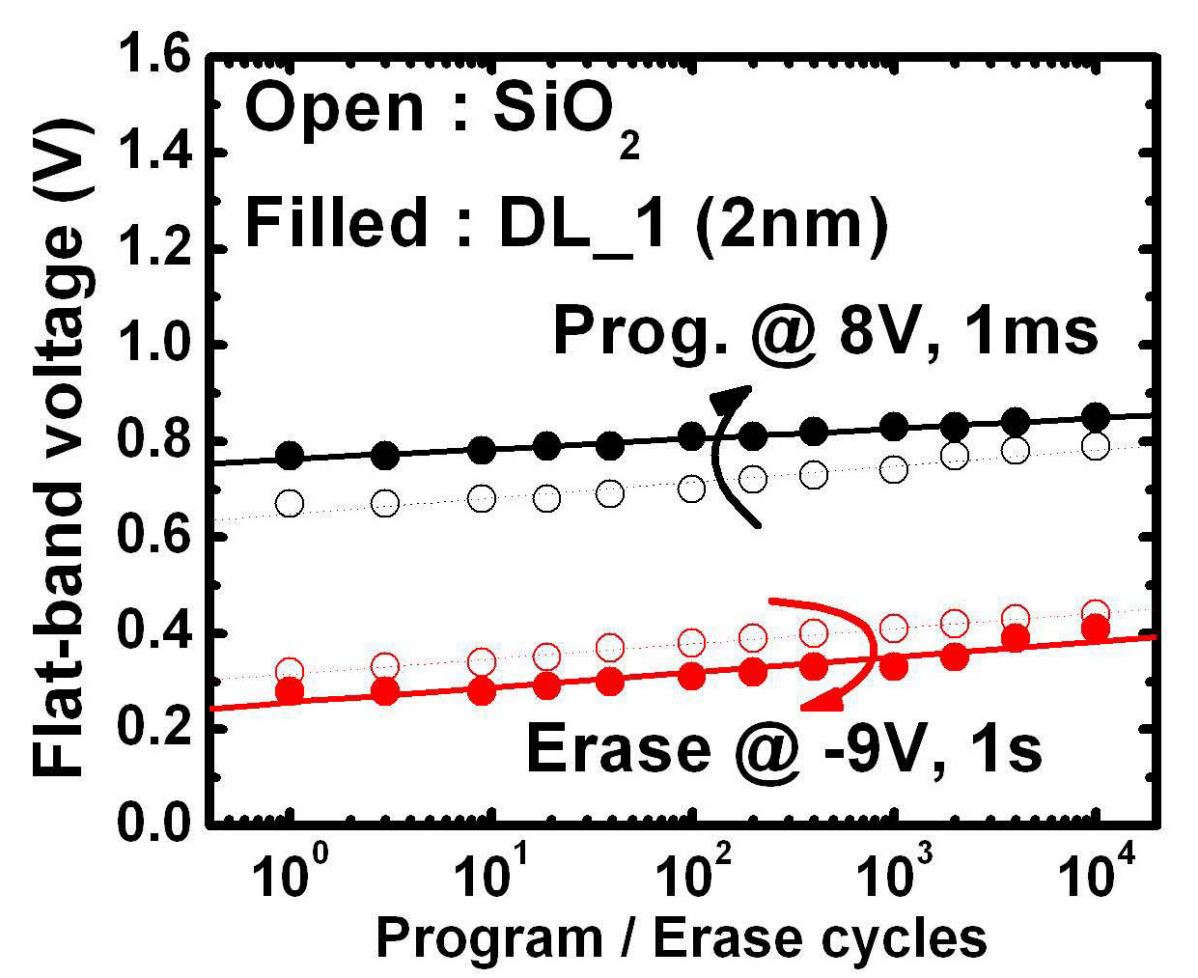
**Endurance characteristics**. The endurance characteristic to 10^4 ^cycles operation for Gd_2_O_3_-NC memories with nanostructure (DL_1 (2 nm)) and single (SiO_2_) tunneling layer. The P/E conditions are 8 V, 1 ms and -9 V, 1 s, respectively.

Based on the retention characteristics, the band diagram at the retention state of the DL_1 (2 nm) sample can be extrapolated as shown in Figure 7a. The lower bandgap of Gd_2_O_3_-NC is surrounded by the higher bandgap of a-Gd_2_O_3_, as mentioned in a previous section. The bandgaps for Al_2_O_3 _and HfO_2 _are 8.7 and 6.1 eV, respectively. Besides, the conduction band offset between Al_2_O_3 _and SiO_2 _is 0.7 eV, while that between Al_2_O_3 _and HfO_2 _is 1.3 eV [24,26]. The band structure of Gd_2_O_3_-NC was proposed using an UV-visible spectrophotometer and by X-ray diffraction spectroscopy [25]. The increased physical thickness of the nanostructure tunneling layer can prevent electron loss from the shallow and deep traps in the Gd_2_O_3_-NC. The higher activation energy of the shallow-trap charge loss is due to thermionic emission of the electrons from the Gd_2_O_3_-NC to the conduction and tunneling back to the Si or Al gate electrode. The lower activation energy of the deep-trap charge loss is due to direct tunneling of electrons from the Gd_2_O_3_-NC to the SiO_2_/Si interface state. In general, the direct tunneling mechanism largely depends on thickness rather than temperature; this is why a lower charge loss rate is observed at a later stage, i.e., the large physical thickness of the nanostructure tunneling layer, as shown in Figure 6a. On the other hand, based on the programming characteristics, the band diagram at the programming state of the DL_1 (2 nm) sample can be extrapolated as shown in Figure 7b. The band bending of the nanostructure tunneling layer when applying the gate voltage could result in electrons tunneling from Si to Gd_2_O_3_-NC. For the DL_1 (2 nm) sample, due to the existence of the low *k *value and the thin SiO_2 _layer, we can estimate that the electric field in the SiO_2 _layer is high. Thus, the electrons will tunnel through the thin SiO_2 _layer via direct tunneling mechanism, and the HfO_2 _layer is no longer a barrier for electrons. Based on this model, the higher P/E speed of the DL_1 (2 nm) sample as shown in Figure 5 can be obtained, especially for the high gate voltage. However, for the thicker HfO_2 _layer (DL_2 (5 nm)), the HfO_2 _layer could be a barrier for electrons when applying the same gate voltage because the electric fields across the tunneling layers become smaller, leading to a low electron tunneling probability. On the other hand, compared with the SiO_2 _tunneling layer, the sample with only the Al_2_O_3 _tunneling layer has lower P/E speed owing to the higher permittivity and thickness of the Al_2_O_3 _layer. Figure 8 shows the endurance characteristics of the Gd_2_O_3_-NC memories. The P/E states exhibit a negligible change after 10^4 ^P/E cycles. This result indicates that the reliability of the Gd_2_O_3_-NC memories is not affected by the nanostructure tunneling layer, and the device could potentially be used in advanced NVMs.

## Conclusions

In this study, we examined the Gd_2_O_3_-NC memories with a nanostructure tunneling layer comprising HfO_2_/Al_2_O_3_/SiO_2_. When compared with devices comprising a single tunneling layer, these NC memories with a nanostructure tunneling layer exhibit a larger *V*_FB _shift and greater data retention because of the band alignment and the increased physical thickness of the tunneling layer. From the retention characteristics, it is observed that the activation energy is 0.13 to 0.17 eV for shallow-trap charge loss and 0.07 to 0.08 eV for deep-trap charge loss. Because the charge loss mechanism for the shallow trap is dominated by thermionic emission, the activation energy is higher than that for the charge loss mechanism of the deep trap, which is dominated by direct tunneling. A band diagram was proposed to completely explain the programming and retention characteristics. In contrast, the endurance characteristics are not influenced by the nanostructure tunneling layer. The Gd_2_O_3_-NC memories with nanostructure tunneling layers could potentially be used in future NVM applications.

## Competing interests

The authors declare that they have no competing interests.

## Authors' contributions

The lead and corresponding author J-CW conceived and designed the experiment, guided this study, carried out the data analysis and theory establishment, and optimized the structure of the manuscript. C-TL participated in the data and theory establishment, guided the detailed experiments, and drafted and wrote the manuscript. C-HC executed the device fabrication and the data measurements, and participated in the data analysis and tabulation of results. All authors read and approved the final manuscript.
